# Body Matters in Emotion: Restricted Body Movement and Posture Affect Expression and Recognition of Status-Related Emotions

**DOI:** 10.3389/fpsyg.2020.01961

**Published:** 2020-08-11

**Authors:** Catherine L. Reed, Eric J. Moody, Kathryn Mgrublian, Sarah Assaad, Alexis Schey, Daniel N. McIntosh

**Affiliations:** ^1^ Department of Psychological Science, Claremont McKenna College, Claremont, CA, United States; ^2^ Wyoming Institute for Disabilities (WIND), University of Wyoming, Laramie, WY, United States; ^3^ Department of Psychology, Scripps College, Claremont, CA, United States; ^4^ Department of Psychology, University of Denver, Denver, CO, United States

**Keywords:** embodied emotion, nonverbal communication, dominance, pride, anger

## Abstract

Embodiment theory suggests that we use our own body and experiences to simulate information from other people’s bodies and faces to understand their emotions. A natural consequence of embodied theory is that our own current position and state contributes to this emotional processing. Testing non-disabled individuals, we investigated whether restricted body posture and movement influenced the production and recognition of nonverbal, dynamic emotional displays in able-bodied participants. In Experiment 1, participants were randomly assigned to either unrestricted or wheelchair-restricted (sitting, torso restrained) groups and nonverbally expressed six emotions (disgust, happiness, anger, fear, embarrassment, and pride) while being videotaped. After producing each emotion, they rated their confidence regarding how effectively they communicated that emotion. Videotaped emotional displays were coded for face, body, and face + body use. Based on naïve coders’ scores, both unrestricted and wheelchair-restricted groups produced emotionally congruent face and body movements and both groups were equally confident in their communication effectiveness. Using videos from Experiment 1, Experiment 2 tested non-disabled participants’ ability to recognize emotions from unrestricted and wheelchair-restricted displays. Wheelchair-restricted displays showed an overall decline in recognition accuracy, but recognition was selectively impaired for the dominance-related emotions of disgust and anger. Consistent with embodied emotion theory, these results emphasize the importance of the body for emotion communication and have implications for social interactions between individuals with and without physical disabilities. Changes in nonverbal emotion signals from body restrictions may influence social interactions that rely on the communication of dominance-related social emotions.

## Introduction

Social interactions rely strongly on nonverbal emotional displays to communicate social emotions, inform others about our feelings, and influence social outcomes (Vosk et al., 1983; Tiedens and Leach, 2004). Such communication can include expressions of basic emotions (e.g., happiness, anger, etc.; Ekman, 1992), as well as more complex social emotions that reflect relative social status (e.g., pride and shame; App et al., 2011; Steckler and Tracy, 2014; Tracy et al., 2015). The literature focuses on the role of the face in emotional communication, but a growing number of studies have demonstrated that the body plays a crucial role in emotion communication as well (Dael et al., 2012). Although some emotions are often communicated *via* facial expressions (e.g., happiness, anger), others are expressed *via* body posture and movement (e.g., pride, embarrassment; App et al., 2011; Tracy et al., 2015).

*Emotional embodiment theory* suggests we use information from our own faces and bodies to understand other people’s emotions as well as our own (Niedenthal et al., 2001). Growing evidence suggests that when we view an emotion displayed by another, the viewer’s ability to recognize, understand, and respond to the emotion relies on the sensory-motor simulation of the observed emotion (Wood et al., 2016). Indeed, over 40 years of research has shown that humans rapidly match the facial expressions of others and that the configuration of their own faces can influence their own emotional states (McIntosh, 1996; Moody and McIntosh, 2006, 2011; Wood et al., 2016). For example, when judging a word for its emotional meaning, people may use their facial muscles to aid the decision (Niedenthal et al., 2009). Further, experimentally manipulating one’s facial expression can affect emotional experience. When facial expressions are restricted (e.g., putting a pen in their mouths), participants are less accurate at identifying others’ emotions (Niedenthal et al., 2001). However, embodiment theory also suggests that information from bodies is also simulated when processing other people’s emotions (Niedenthal et al., 2001).

One less explored implication of embodied theory is that emotional perception is an interactive process and that the interaction between emotion experience and the physical display of emotion is reciprocal. That is, a person’s state, which includes current inputs from body position, influences the simulation process and the perception of others’ emotions. Indeed, it is known that people not only physically display their subjective emotional state but also their emotional display affects their subjective emotional state (see McIntosh, 1996). This ability to use physiological changes and to re-experience one’s somatic responses plays an important role in emotional processing (Niedenthal, 2007; Halberstadt et al., 2009). Further, the body conveys emotion information that is different from the face and can even modulate emotional information conveyed by the face (Aviezer et al., 2008; App et al., 2012; Abo Foul et al., 2018). Studies show that people’s own bodies influence the perception of their own as well as others’ emotional states (McIntosh, 1996; Niedenthal et al., 2005; Reed and McIntosh, 2008; Wilbarger et al., 2011).

Thus, a consequence of this theory is that if either face or body use is impeded, it will have implications for their perceptions, experiences, and communications of emotions (de Gelder, 2006; App et al., 2011; Steckler and Tracy, 2014; Tracy et al., 2015; Moody et al., 2018). A study by Wallbott and Giessen (1986) confirmed that viewing whole body videos of actors portraying emotional scenarios lead to more accurate emotion recognition than audio-only recordings of the same scenarios. Importantly, Moody et al. (2018) demonstrated rapid, emotionally congruent, whole-body responses from static emotional faces, indicating that the participants simulated the viewed facial emotion using their whole bodies. When judging changes in other people’s body postures, input from one’s own emotional body posture selectively influences perceptions of another person’s emotional body posture but not neutral postures (Wilbarger et al., 2011). Nonetheless, little work has explored whether movement restrictions of the body has similar consequences for emotional processing as it does for the face and whether such constraints differentially affect emotions preferentially conveyed *via* the body.

Although some emotions are communicated primarily *via* the face and others rely on the body, individual emotions are associated with specific social functions (Tracy and Robins, 2007; App et al., 2011). For instance, emotions conveying status relations between individuals include anger, disgust, and fear that are associated with facial expressions, as well as embarrassment, guilt, pride, and shame that are associated with bodily expressions (Coulson, 2004; Tracy and Robins, 2007; App et al., 2011; see also Atkinson et al., 2004). Emotional body-expressions are important in conveying social hierarchical information and the degree to which they signal relative social status (Steckler and Tracy, 2014). Generally, more erect postures are associated with higher status and dominance, although context can affect this interpretation (Hall et al., 2005). Actors asked to portray various emotions often used a collapsed body posture for shame or sadness and used body expression more than facial movement to display pride (Wallbott, 1998). Across studies and cultures, pride appears associated with a low intensity smile and a variety of different body positions including expanded posture, arms akimbo on hips or arms raised straight above the head with the hands (Wallbott, 1998; Tracy and Robins, 2007; Tracy and Matsumoto, 2008). Given that emotions have different social functions and differential reliance on face vs. body expression, the restriction of body movement may not affect the processing of emotions in the same way (de Gelder, 2006; App et al., 2011; Steckler and Tracy, 2014; Tracy et al., 2015).

### Current Study

Relatively little research has explored the role of the body on emotional communication between individuals. Moreover, few studies have considered how the prevention of body movement might differentially disrupt the production and recognition of specific emotions. Here, we examine how restricted body movement and posture influences emotional expression and recognition. Given that experimentally preventing or enhancing emotional facial expressions can influence emotional experience of basic emotions (i.e., anger, fear, happiness, and sadness; Ekman, 1992; Jack et al., 2014), constraints on body movement and posture may also affect the sensorimotor processing or simulation of emotions, especially more complex, body-related emotions (e.g., embarrassment and pride). Such constraints could influence both an observer’s perception and nonverbal communication of emotions. They may also differentially affect the recognition of emotions by changing the visual cues that distinguish them (Wallbott, 1998; Sawada et al., 2003; Gross et al., 2010). Moreover, body and posture constraints may affect more than just body-related emotions because they change the implied social dominance between individuals. Although disgust is best conveyed by the face and pride is best conveyed by the body, both emotions indicate one person’s superior standing compared to another. In other words, emotions that convey social status may supersede the body-related vs. face-related emotion distinction because body constraints do more than just restrict movement.

In two studies we extend work documenting the body’s role in emotion displays (e.g., de Gelder, 2006; Tracy and Robins, 2007; App et al., 2011). In Experiment 1, we examined the role of body movement and postures restrictions on nonverbal emotion communication for non-disabled participants. In Experiment 2, we determined whether these constraints affected emotion recognition in non-disabled participants. This research is a first step in understanding the role of embodiment in the communication of social emotions and its implications for how nonverbal emotional displays may influence social interactions between non-disabled and physically disabled individuals.

## Experiment 1: Emotion Production

The purpose of Experiment 1 was to determine whether current body inputs affected non-disabled participants’ nonverbal expressions of emotions and their confidence in the efficacy of communicating emotions. In addition, the experiment replicated previous studies indicating specific face and body preferences for nonverbal emotional communication (App et al., 2011). To examine the effects of body and posture constraints, we compared the expression of six emotions for two groups that differed in their mobility restrictions: (1) an “unrestricted” group in which participants stood while communicating emotions without mobility restrictions and (2) a “wheelchair-restricted” group in which participants sat in a wheelchair with an elastic band tied around the torso, thereby restricting trunk movement. The unrestricted condition provided a reliable baseline to which we could compare performance in the movement-restricted condition. In Part 1, participants communicated six emotions nonverbally to a mannequin while being videotaped; after expressing each emotion, they rated their confidence in successfully communicating the emotion. In Part 2, participants indicated whether they preferred face or body channels to optimally communicate each emotion.

Extensions of embodiment theory would predict that current physical inputs should affect the communicator’s production of nonverbal, emotional displays as well as the communicator’s confidence in the effectiveness of those displays, especially for those emotions best conveyed by the body. Therefore, compared to the unrestricted group, the wheelchair-restricted group should show reduced use of the body and increased use of the face during emotional production, especially for emotions optimally expressed by the body. The postural and movement differences between groups should also differentially affect status emotions. Further, if current body inputs were used for evaluating one’s own communication, then the wheelchair-restricted group may be less confident in communicating body-based emotions. Of interest is whether confidence in emotion communication was affected by current body constraints.

### Method

#### Participants

Forty-nine participants (33 female, mean age = 19.6 years) received partial course credit in introductory level psychology courses for their participation. They were randomly assigned *via* a computer program to one of two groups: the unrestricted group (*n* = 21) and the wheelchair-restricted group (*n* = 28). In the unrestricted group, participants stood 3 ft from a seated mannequin. In the movement restricted “wheelchair-restricted” group, participants sat in a wheelchair with their chests strapped into the wheelchair by a stretch athletic band around the lower rib cage to stabilize and restrict movement of the trunk. Straps reduced torso motion shown to be important for conveying emotional state (e.g., Wallbott, 1998; Atkinson et al., 2004). Participants were still able to use their arms and limited torso adjustments to express emotions.

#### Stimuli and Apparatus

Participants directed their emotions toward a life-size mannequin with a soft, gray, fabric exterior and no definitive facial features. The mannequin was seated in a chair in front of the participants and dressed in a casual, gender-neutral outfit, including a sweat suit and baseball hat. Participants were asked to think of someone they knew and to address the mannequin as if it were that person, whether it was a friend, relative, or romantic partner. The mannequin was addressed as the chosen person throughout the experiment. The mannequin’s lack of facial features provided participants with a consistently neutral response to their emotion production.

To record participant’s nonverbal emotional displays, two video cameras were used to film the facial and body expressions of the participants; one was placed behind the mannequin to provide a face-front view of the participants and another camera was placed to the side of the participants to provide a side view that showed both the expresser and the mannequin.

To quantify the relative degree of participants’ face and body use during the communication of each emotion, we modified a coding scheme used in App et al. (2011) to assess the amount of emotionally congruent movement in the face and the body as recorded from the front and side view videos. Emotion congruence was defined as using non-random, systematic movements to convey each emotion and followed classification procedures described in App et al. (2011). More specifically, coders examined videos for face and body movements that the literature has confirmed to be associated with, or congruent with, each of the emotions used in this study (Atkinson et al., 2004; Moody and McIntosh, 2006; App et al., 2011). For example, for a happy condition trial, a congruent emotional display would indicate the presence of upward movements in the eyes and cheeks resulting from zygomaticus muscle activation in the face (Moody and McIntosh, 2006) and/or the lifting of arms or jumping up and down (Atkinson et al., 2004). However, if the participant displayed a furrowing of the forehead resulting from corrugator muscle activation that is typically associated with anger displays, then this movement would be considered incongruent with the emotion communicated.

For each participant, condition, and trial, video data were coded separately from both the front and side-view videos. In both views, the face and the body were visible. Face and body movements were coded independently for each of three categories that are reflected in the levels of the statistical analyses: (1) face alone – emotionally congruent face movement without simultaneous body movement; (2) body alone – emotionally congruent body movement without simultaneous face movement; and (3) face + body – emotionally congruent simultaneous face and body movement. Three scorers, who were naïve to the predictions of the experiment, rated movement for each of these three categories using a scale ranging from 0 [none: no emotionally congruent movement (i.e., no use of muscles specific to of the communicated emotion)] to 1 (some emotionally congruent movement: movement indicated sufficient information for some level of emotion congruence but not a full match) to 2 (full match of expected emotionally congruent movement). Scores from these naïve scorers indicated agreement on 92% of trials overall, with 97% agreement for the unrestricted group and 87% agreement for the restricted wheelchair group. The average use of emotionally congruent face, body, or concurrent face and body movements was calculated for each participant over two views and three trials for each of the six emotions.

### Procedure

#### Part 1: Emotion Production

Tested individually, participants nonverbally communicated six different emotions (anger, disgust, fear, happiness, pride, and embarrassment) to the mannequin as effectively as possible. For each participant, a computer program randomly selected group assignment. The experiment began with practice trials in which participants produced two emotions not used in the experimental trials (surprise and sadness). Experimental trials followed in which the participant pressed a computer key to begin the trials and one of the six emotion words appeared on the computer screen. The participant then expressed the emotion to the mannequin for 4 s. Following each expression trial, participants were prompted to enter number on a scale of 0 (“not confident at all”) to 4 (“very confident”) to indicate their confidence in how well they were able to communicate that emotion. Each of the six emotions was produced three times in random order for a total of 18 production trials and 18 confidence ratings. To reduce any potential influences of experimenter presence on emotion production, the experimenter remained outside the testing room after practice trials.

#### Part 2: Preferences for Using the Face or Body to Communicate Specific Emotions

After completing the emotion production portion of the experiment, participants remained in their unrestricted or wheelchair-restricted states. An emotion word cue appeared on the computer screen and they indicated *via* a key press whether they would *typically* (i.e., in the everyday world) use their face or their body to nonverbally express that emotion. They provided a single face/body preference for each emotion; emotion order was randomized.

### Results and Discussion

#### Preferences for Face vs. Body Use When Conveying Emotions

**Figure 1:**
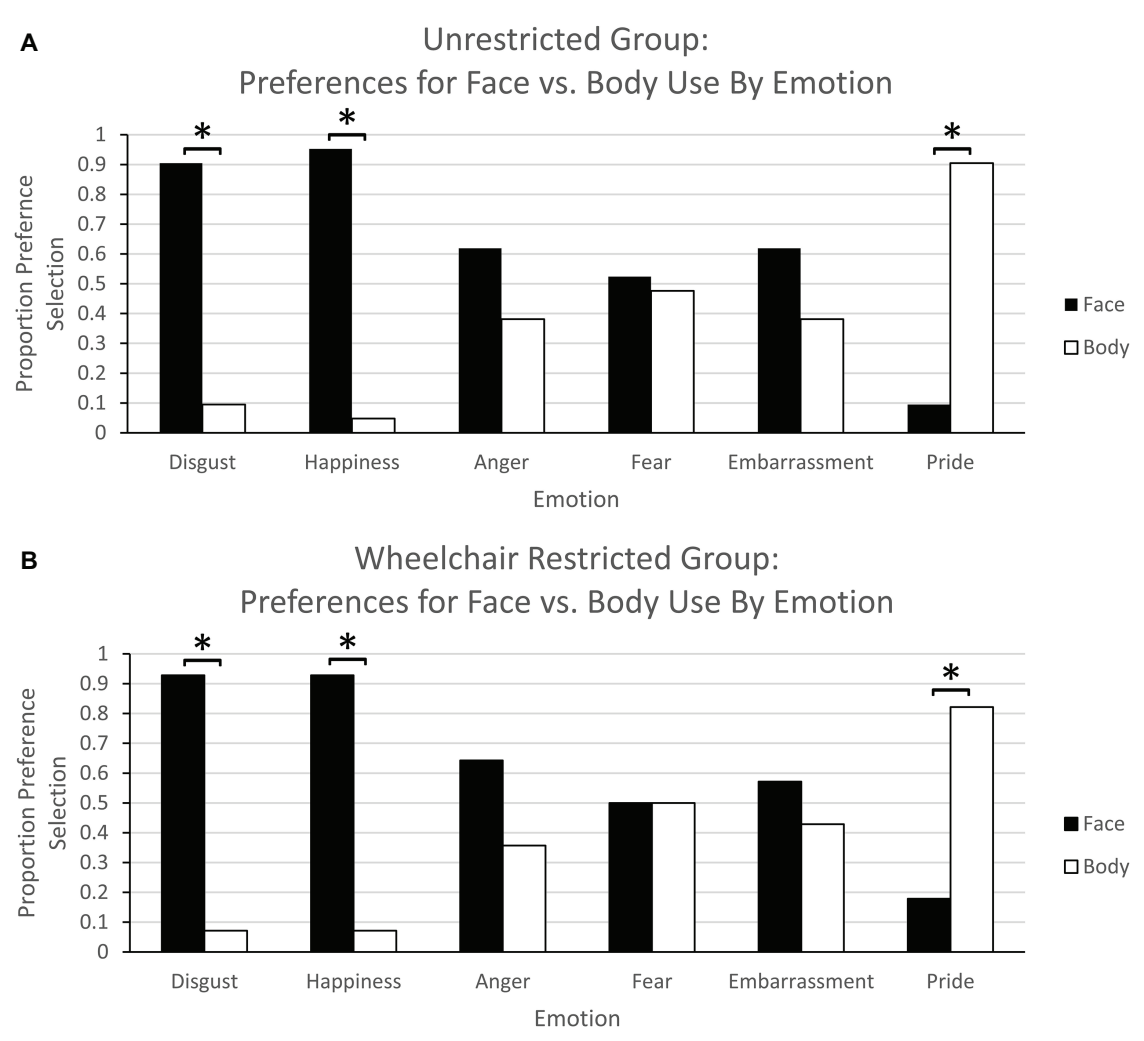
Experiment 1 – Proportion preference selection for face vs. body use for emotion production: **(A)** unrestricted group, **(B)** wheelchair-restricted group. There were no group differences. For each emotion, stars indicate significant preference differences for using either the face or the body.

The purpose of this analysis was to replicate findings by App et al. (2011) and to confirm that the participants in our study endorsed similar preferences for using the face or body to express each emotion. The results confirm that preferences to use the face vs. the body to express emotions differs across emotions and show a similar pattern to those reported in App et al. (2011). For each group and emotion, the frequency of preferred face vs. body use was tabulated. To document differential preferences for face or body use when communicating the six emotions, chi-square tests were conducted for face/body preferences for each emotion and group using frequency data. There were no group differences overall in body/face expression preferences for any emotion [*χ*^2^(1) < 0.70, *p* > 0.71]. Regardless of group, face/body preference largely replicated previous findings (App et al., 2011; Figures 1A,B). Participants preferred to use the body to communicate *pride* [unrestricted group: *χ*^2^(1) = 13.762, *p* < 0.0001; wheelchair group: *χ*^2^(1) = 11.57, *p* < 0.001]. They preferred to use the face for *disgust* [unrestricted group: *χ*^2^(1) = 13.76, *p* < 0.0001; wheelchair group: *χ*^2^(1) = 20.57, *p* < 0.0001] and *happiness* [unrestricted group: *χ*^2^(1) = 17.19, *p* < 0.0001; wheelchair group: *χ*^2^(1) = 20.57, *p* < 0.0001]. Preferences were more divided for face and body use when expressing *anger* [unrestricted group: *χ*^2^(1) = 1.19, *p* = 0.28; wheelchair group: *χ*^2^(1) = 2.29, *p* = 0.13], *fear* [unrestricted group: *χ*^2^(1) = 13.76, *p* = 0.83; wheelchair group: *χ*^2^(1) = 0.000, *p* = 1.000], and *embarrassment* [unrestricted group: *χ*^2^(1) = 1.19, *p* = 0.28; wheelchair group: *χ*^2^(1) = 0.57, *p* = 0.45]. These preferences for each emotion (summarized in Table 1) establish a baseline indicating whether people typically rely on the face or body to nonverbally communicate specific emotions: The face is preferred to convey disgust and happiness, the body is preferred to convey pride, and we found no significant difference in preference for face or the body conveys anger, fear, and embarrassment.

**Table 1:** Face vs. body use preferences for emotion production.

	Disgust	Happiness	Anger	Fear	Embarrassment	Pride
Face	x	x	x	x	x	
Body			x	x	x	x

Despite the fact that participants were either unrestricted or wheelchair-restricted when providing face‐ or body-use preferences, the similarity of responses for the two groups suggests that these preferences were not influenced by current body inputs and rather were based on functional, and/or experiential mechanisms. These data also suggest that the recognition of emotions that rely most on the body (tested in Experiment 2) – pride followed by embarrassment, fear, and anger – may be most affected by restricting torso movement in a wheelchair.

#### Emotion Production: Emotion-Specific Influences of Restricted Body Posture and Movement

For all analyses of variance (ANOVAs) using ordinal data in both experiments, we report comparisons with Huyhn-Feldt adjustments for sphericity, which is proposed as a sufficient condition for the *F*-tests to be valid for ordinal data (Stiger et al., 1998). We applied the Dunn–Šidák correction to all simple effects analyses to correct for multiple comparisons. Alpha was set at the 0.05 level.

To assess whether posture and body constraints differentially influenced the production of emotions, we conducted a mixed model ANOVA with the between-subject factors of group (2: unrestricted and wheelchair-restricted) and within-subject factors of face/body use [3: face, body, face + body (i.e., concurrent face and body)] and emotion (6: disgust, happiness, anger, fear, embarrassment, and pride) using emotion-congruent movement scores provided by three observers. The unrestricted and restricted groups did not differ in the presence of emotionally congruent movement [group effect: *F*(1,47) = 0.42, *p* = 0.52, *η_p_*^2^ = 0.01; *M*_unrestricted_ = 1.33, *SE* = 0.08; *M*_wheelchair_ = 1.27, *SE* = 0.07].

**Figure 2:**
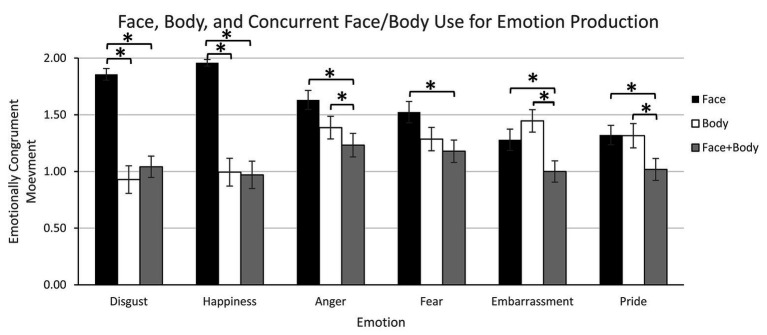
Experiment 1 – Face, body, and concurrent face/body use for emotion production. Error bars indicate standard error. Stars indicate significant differences between face, body, and concurrent face + body use.

Also, the presence of emotionally congruent movements did not differ across emotions [emotion effect: *F*(4.98, 234.13) = 1.19, *p* = 0.31, *η_p_*^2^ = 0.03], regardless of group [group × emotion interaction: *F*(4.98, 234.13) = 0.97, *p* = 0.44, *η_p_*^2^ = 0.02]. The interaction between group and face/body use was not significant at the 0.05 level, [*F*(1.26, 59.05) = 2.78, *p* = 0.06, *η_p_*^2^ = 0.06]. However, the interaction between emotion and face/body use indicated differential use of the face, body, and concurrent face and body (face + body) across emotions [*F*(6.35, 298.66) = 14.88, *p* < 0.0001, *η_p_*^2^ = 0.24]. The emotion by face/body use interaction was consistent with the preference data, indicating that the face alone is used to express disgust and happiness more than the body alone or their combination; the face and body alone, more than their combination, are used to express anger, embarrassment, and pride; and the face is used more than the face + body combination to express fear (Figure 2).

Direct comparisons showed that for disgust the face was used more than the body or both concurrently (i.e., face + body; *p* < 0.0001) and there was no difference for body vs. face + body use (*p* = 0.31). Similarly, for happiness, the face was used more than the body or face + body (*p* < 0.0001) and there was no difference for body vs. face + body use (*p* = 0.58). However, for anger, face and body use did not differ significantly (*p* = 0.15), but both the face (*p* < 0.0001) and the body (*p* = 0.02) were used more than face + body. Similarly for fear, face and body use did not differ significantly (*p* = 0.16) but the face was used more than face + body (*p* < 0.0001), and body use was similar to face + body (*p* = 0.47). For embarrassment, face and body use did not differ significantly (*p* = 0.55), but both the face (*p* < 0.003) and the body (*p* < 0.0001) were used more than face + body. Finally, for pride, face and body use did not differ (*p* = 1.00) and both the face (*p* < 0.002) and the body (*p* < 0.0001) were used individually more than together (face + body). Further, although concurrent use of the body and face (face + body) did not differ across emotions (*p* > 0.64), the face was used significantly more for disgust and happy than anger, fear, embarrassment, and pride (*p* > 0.02), it was used more for anger than embarrassment (*p* < 0.005); other face comparisons were not significant. Finally, significantly greater body use was observed for anger and embarrassment than disgust and happiness (*p* < 0.03). The group by emotion by face/body use interaction was in the predicted direction but was not significant at the alpha = 0.05 level [*F*(6.35, 298.66) = 1.96, *p* = 0.06, *η_p_*^2^ = 0.04].

Because the groups were created by random assignment, they had different sizes (unrestricted = 1 vs. wheelchair restricted = 28). To demonstrate that unequal group size did not affect the results, we randomly selected 21 participants from the larger group and reran the above analysis. The results for the equal-sized groups were highly comparable, but the group × emotion × face/body use interaction was now significant (*p* = 0.05)^1^.

#### Confidence in Effective Emotion Communication

**Figure 3:**
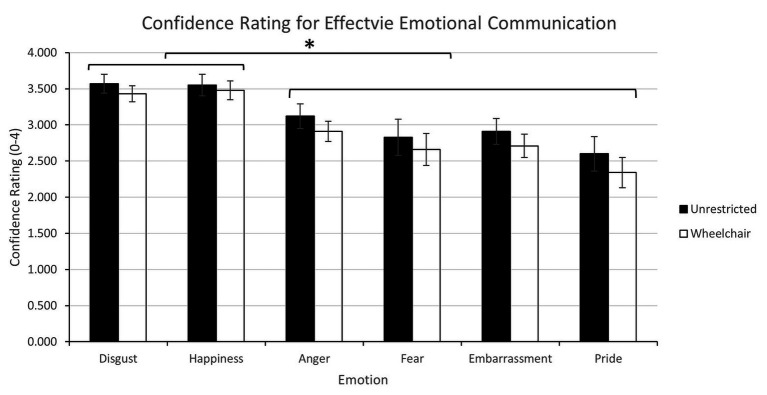
Experiment 1 – Confidence ratings for effective emotion communication overall. Error bars indicate standard error. Stars indicate significant confidence differences across emotions: confidence for disgust and happiness was greater than for the other emotions.

To examine the effects of current bodily constraints on confidence in emotional expression, a mixed model ANOVA with the between-subject factors of group (2: unrestricted and wheelchair) and within-subject factors of emotion (6: disgust, happiness, anger, fear, embarrassment, and pride) was conducted on confidence ratings. Ratings should differ by group if current body inputs influenced confidence in emotional communication but not if previous experience were more important because non-disabled participants have similar *prior* experiences. A significant main effect for emotion [*F*(4.31, 202.62) = 13.55, *p* < 0.0001, *η_p_*^2^ = 0.22] indicated greater confidence when communicating disgust and happiness than anger, fear, embarrassment, and pride (*p* < 0.001). Of interest, the posture and body restrictions imposed by the wheelchair did not influence confidence ratings, as indicated the lack of a group effect [*F*(1, 47) = 1.48, *p* = 0.23, *η_p_*^2^ = 0.03] or the group by emotion interaction [*F*(4.31, 202.62) = 0.08, *p* = 1.00, *η_p_*^2^ = 0.002; Figure 3].

In sum, replicating prior research (e.g., App et al., 2011), Experiment 1 first established preferences for the selective use of the face and body for specific emotions. Preference data confirm that non-disabled individuals do not prefer to use the face to convey all emotions and that they would use the face and body differentially depending on the emotion. Participants indicated that they preferred to use the face to best convey disgust and happiness; they were divided in their preference to use the face or body to express anger, fear, and embarrassment; and they preferred to use the body to convey pride.

Next, Experiment 1 examined how constraining body posture and movement might influence the nonverbal communication of these emotions in non-disabled participants. Comparing the nonverbal, physical production of emotions between the unrestrained and wheelchair-restrained groups, movement restriction in non-disabled individuals did not strongly alter the production of emotionally congruent movement. Further studies are needed with physically disabled individuals to more fully explore potential differences in the relative use of the face and body in emotion production. Further, no group differences emerged between the unrestricted and wheelchair group’s confidence in their effective communication of the emotions. Prior emotional experience for our non-disabled participants who are not typically constrained in their movements appears to have more influence on their confidence in emotional communication than current body inputs. Nonetheless, posture and torso constraints had an overall influence emotion production, which may convey differential nonverbal emotion signals to observers. Thus, posture and movement constraints may also influence the reception of emotional displays. We investigate this issue in Experiment 2.

## Experiment 2: Emotion Identification

Experiment 2 investigated whether the expression of emotions by individuals with physical constraints affects the recognition of nonverbal emotional displays. Using a sample of emotion production videos from each group in Experiment 1, we assessed whether constraints on body movement and posture influenced the accuracy of emotion identification overall or for specific emotions.

### Method

#### Participants

Participants were 22 students (10 females; age: *M* = 19.3 years, *SD* = 1.14) who received partial course credit in lower level psychology courses. None had participated in Experiment 1.

#### Stimuli and Apparatus

A subset of video recordings was selected from Experiment 1 to limit the duration of Experiment 2. From video clips recorded in Experiment 1, two male and two female participant video clips were selected at random for each of the six emotions. Video clips were edited to remove the sound and have a 4-s duration. A total of 48 video clips were used: 2 males × 2 females × 2 conditions (unrestricted and wheelchair) × 6 emotions. All video clips were emotional displays recorded from the side view so that the observer could see the participant interacting with the mannequin. Stimuli were presented on a 21-inch computer monitor with E-prime 2.0 pro software (Psychological Software Tools, Pittsburgh, PA).

### Procedure

Participants were tested individually and were asked to identify emotions presented in silent video clips. For each emotion recognition trial, a 4-s emotion video was played twice on a computer monitor, with a 500 ms pause between presentations. A numbered list of emotions (1 = *anger*, 2 = *sympathy*, 3 = *embarrassment*, 4 = *love*, 5 = *disgust*, 6 = *fear*, 7 = *happiness*, 8 = *sadness*, 9 = *pride*, plus 0 = *other*) appeared and participants indicated the emotion presented in the video by pressing the number key associated with the corresponding emotion word. The list included three additional emotions (sympathy, sadness, and love) as well as an “other” category to reduce inflated accuracy rates due to forced choice (Russell, 1993; Frank and Stennett, 2001). Participants then rated their confidence that their response was correct on a scale from 1 (not at all confident) to 5 (extremely confident). After two practice trials, participants completed three blocks of 48 experimental trials (6 emotions × 2 male × 2 female × 2 conditions; each stimulus was presented 3 times) for a total of 144 trials; within each block, trials were presented in random order. A brief break was provided between each block.

### Results

For participant, emotion, and condition, mean proportion accuracy was calculated for emotion identification; correct responses were recorded when the selected emotion matched the viewed emotion. Mean confidence ratings were also calculated for each participant, emotion, and condition. For all ANOVAs using ordinal data in this paper, we report comparisons with Huyhn-Feldt adjustments for sphericity (Stiger et al., 1998). The Dunn–Šidák correction was applied to all simple effects analyses to correct for multiple comparisons. Alpha was set at the 0.05 level.

#### Emotion Identification Accuracy

**Figure 4:**
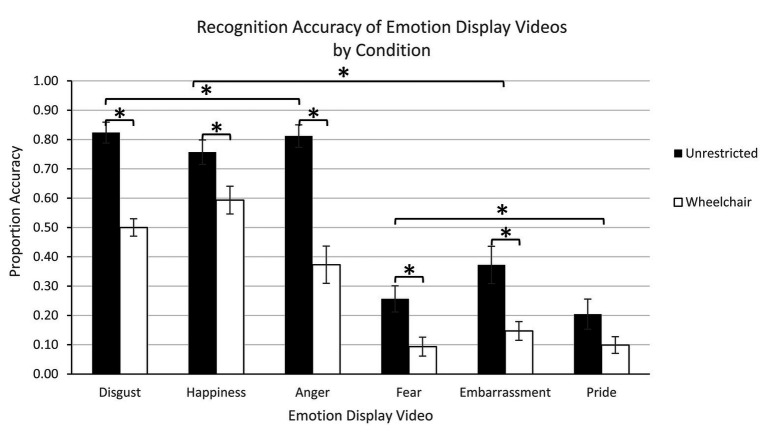
Experiment 2 – Recognition accuracy of emotion display videos by condition. Error bars indicate standard error. Stars indicate significant differences between conditions and emotions.

To determine if emotional displays under restricted posture and body movement conditions affected emotion recognition, we conducted a within-subjects ANOVA with factors viewed condition (2: unrestricted and wheelchair) and emotion (6: anger, disgust, fear, happiness, pride, and embarrassment) using mean proportion accuracy data. Emotions expressed in unrestricted conditions (*M*_unrestricted_ = 0.46, *SE* = 0.02) were more accurately identified than emotions expressed in wheelchair-restricted conditions (*M*_wheelchair_ = 0.30, *SE* = 0.02) condition: [*F*(1,20) = 66.77, *p* < 0.0001, *η_p_*^2^ = 0.77]. The main effect of emotion indicated that some emotions were recognized more accurately than others [*F*(5, 100) = 63.62, *p* < 0.0001, *η_p_*^2^ = 0.76]. Simple effects comparisons (all values of *p* > 0.0001) revealed that accuracy was greatest for disgust (*M* = 0.66, *SE* = 0.02), happiness (*M* = 0.68, *SE* = 0.03), and anger (*M* = 0.59, *SE* = 0.04) compared to fear (*M* = 0.18, *SE* = 0.03), embarrassment (*M* = 0.26, *SE* = 0.04), and pride (*M* = 0.15, *SE* = 0.02); accuracy did not significantly differ between anger, happiness, and fear or across fear, embarrassment, and pride (all values of *p* > 0.51). Differences across emotions regarding the impact of posture and body movement on emotion recognition are reflected in the interaction between condition and emotion [*F*(5,100) = 4.23, *p* < 0.002, *η_p_*^2^ = 0.17; Figure 4]. Simple effect analyses revealed greater accuracy for unrestricted over wheelchair-restricted conditions for disgust (*p* < 0.0001), happiness (*p* = 0.02), anger (*p* < 0.0001), fear (*p* = 0.01), and embarrassment (*p* < 0.003) but not pride (*p* = 0.14). Although the difference between the unrestricted and restricted conditions for pride is not significant, we believe this one lack of difference in condition significance can be explained by the fact that pride was less recognizable in the unrestricted condition. The accuracy for wheelchair conditions for body-related displays of fear, embarrassment, and pride all showed low levels of accuracy.

#### Confidence for Emotion Recognition

**Figure 5:**
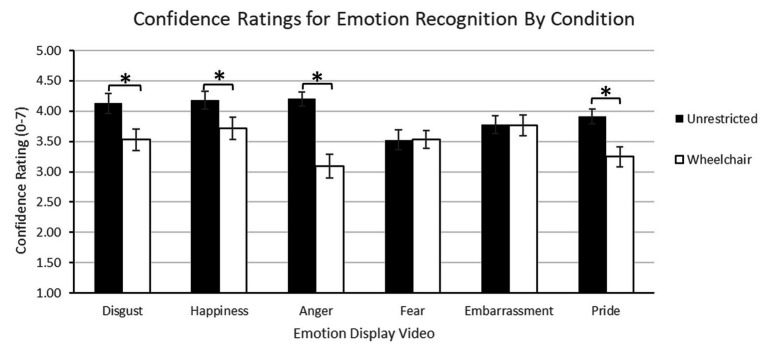
Experiment 2 – Confidence ratings for emotion recognition by condition. Error bars indicate standard error. Stars indicate significant condition differences.

For confidence data, a within-subjects ANOVA was conducted for the factors condition (2: unrestricted and wheelchair) and emotion (6: disgust, happiness, anger, fear, embarrassment, and pride). Participants indicated greater confidence in emotion identification in unrestricted (*M*_unrestricted_ = 3.95, *SE* = 0.10) than wheelchair-restricted (*M*_wheelchair-restricted_ = 3.48, *SE* = 0.13) conditions [*F*(1, 20) = 21.45, *p* < 0.0001, *η_p_*^2^ = 0.52]. The significant emotion effect [*F*(5, 93.59) = 3.82, *p* = 0.004, *η_p_*^2^ = 0.16] showed that participants were equally confident for disgust, happiness, anger, and embarrassment (all values of *p* > 0.54) but significantly more confident in recognizing happiness compared to fear (*p* = 0.007) and pride (*p* = 0.002). This effect was mediated by the significant condition by emotion interaction [*F*(6, 100) = 7.02, *p* < 0.002, *η_p_*^2^ = 0.26; Figure 5], further indicating that posture and body-movement restrictions differentially affected participant’s confidence in identifying specific emotions. Generally, participants had greater confidence for face-related compared to body-related emotions. Simple effect analyses confirmed that participants were more confident in their identification of unrestricted over wheelchair-restricted displays for disgust (*p* < 0.006), happiness (*p* = 0.02), anger (*p* < 0.0001), and pride (*p* < 0.0001). Unlike accuracy data, confidence between conditions did not differ for fear (*p* = 0.98) or embarrassment (*p* = 0.91). Interpreting this pattern of results in the context of status-related classifications of emotion from the literature, the greatest confidence differences between unrestricted vs. wheelchair-restricted conditions occurred for the dominant status emotions of anger, pride, and disgust, and the least confidence differences in recognizing the low-status emotional displays of fear and embarrassment.

In sum, emotion recognition accuracy and confidence were strongly influenced by restricting posture and body movement in nonverbal displays. Recognition accuracy and performance confidence declined disproportionately for dominant emotions expressed by non-disabled people in wheelchairs. Conversely, accuracy and confidence for subordinate emotions of fear and embarrassment were affected less by bodily restrictions. Clearly, the high-confidence ratings for effective emotional communication of Experiment 1’s non-disabled but wheelchair-restricted group were not supported by the emotion recognition data.

## General Discussion

This study investigated the contributions of body posture and movement to the production and recognition of nonverbal emotion displays. A less evaluated implication of embodied emotion theory is that emotional experience is bidirectional: Another person’s body induces emotional simulation in the observer’s body and, at the same time, the current inputs from the observer’s body or state contribute to the emotional processing. In this view, a person’s current body inputs may affect the production of nonverbal displays of emotion. In Experiment 1, non-disabled participants were randomly assigned to either the unrestricted or the wheelchair-restricted group. Although we expected the wheelchair-restricted group to use the face more to express body-related emotions because body posture and movement was constrained, we investigated whether these constraints had a general effect for all emotions or whether it influenced some emotions more than others. In Experiment 2, we used emotion production videotapes from Experiment 1 to assess emotion recognition performance from unrestricted and wheelchair-restricted emotional displays.

To confirm that the communication of specific emotions differentially relies on use of the face vs. the body, in Experiment 1 we asked participants to express their preference for face and body use for six different emotions. Consistent with prior literature, we found that preferred use of the body and face differed across emotions, with status-oriented emotions often expressed using the body (e.g., App et al., 2011; Tracy et al., 2015). Participants preferred to use the face to express happiness and disgust, the body to express pride, and there were no significant differences in preference for use of face or both to express anger, fear, or embarrassment. In addition, we found no differences between unrestricted and wheel-chair restricted groups for stated face vs. body use preferences, suggesting that these preferences are not based on current body input. Instead they appear to rely on past experience, socialization (i.e., what configuration has been taught or is typically displayed for that emotion), or a more evolved response.

One primary focus of the study was to assess how restrictions of body posture and movement might affect the relative use of the face and body to nonverbally communicate emotions. An examination of emotionally congruent face and body movements revealed that posture and movement restrictions did not strongly change their presence in this non-disabled sample, although coder’s notes indicated that it affected the size of the movements.

Further, posture and movement restrictions did not affect participants’ confidence ratings as to how well they communicated the emotions. They were equally confident as the unrestricted group that they had successfully communicated each emotion. This was particularly notable for emotions relying on body movement and posture. Current body inputs did not influence non-disabled participants’ perceptions of emotional competency when they were restrained. Instead, like their verbal channel preferences, their confidence ratings appeared to be based on a culmination of past experiences. This suggests that people are not aware of the nonverbal changes that they experience when body posture and movement are constrained to a wheelchair. In light of embodied emotion theory, body restrictions should have altered emotional experience. Contrary to these predictions, we found that despite actual physical emotion production changes, feedback from the expresser’s body was disregarded, ignored, or discounted. Instead of relying on current body inputs, people’s conceptions of emotional competency appeared to be built from experiences/inputs acquired over time.

Nonetheless, body movement restrictions and their corresponding alterations of emotional visual cues did influence participants’ ability to recognize nonverbal displays of emotions. In Experiment 2, participants recognized emotions based on the videos from Experiment 1. Emotion recognition accuracy was impaired for videos of emotions expressed by the wheelchair-restricted individuals. In addition to a large, overall decrement in recognition for wheelchair-restricted displays of emotion, the emotions of disgust and anger were particularly affected. It is important to note that the recognition of all social status emotions is impeded when the expresser is sitting and restrained. Instead, these constraints specifically affect the displays of social dominance emotions. Following embodiment theory, viewing expressers who are seated and constrained may affect emotional simulation and decrease recognition of the emotion in the viewer because it may be more difficult to feel dominant, which is associated with the expression of disgust and anger.

This study represents a first step in establishing how the body and its posture influence the perception and communication of emotions in non-disabled individuals. Our goal was to gain insight into emotional communication issues for people with physical disabilities, restricting them to wheelchairs that constrain both body movement and posture. Although the participants in this laboratory study did not have physical disabilities and we simulated disability by constraining participants to wheelchairs, our goal was to investigate some previously untested implications of embodiment theory and to provide baseline data as to how similar kinds of restrictions influence abled-bodied individuals. Future studies will examine emotional perception and communication for individuals with physical disabilities.

### Implications for Embodiment Theory and Social Interactions

The results of this study have implications for embodiment theory as well as for social interactions. First, current bodily inputs do not affect confidence in emotion displays in a way consistent with actual changes in the efficacy of displays. Generally, people may not be fully aware of situational factors that have an impact on the efficacy of their display. Not surprisingly, in the case of physical restraints that affect body movement and posture, it is the expression of body-involved emotion displays that are most affected.

Differences in emotion displays associated with constrained body movement also may influence those interacting with that person. Specifically, they influence the perception and recognition of dominant emotions. These results have implications for how people might establish dominance in group interactions – unrestricted displays of dominant emotions are interpreted more accurately. People expect those high in power to show erect and open postures, upward tilt of the head, and touching behavior (Carney et al., 2005; Hall et al., 2005). However, because body-related emotions are also associated with conveying relative social status, it is important to consider how such constraints may affect social perception between individuals in work and other settings who are establishing relative social power. Another related factor is the lower relative position of people in sitting positions. A number of studies have shown that relative height between individuals involved in social interactions influences social outcomes and perceived social power (Schwartz et al., 1982;Schubert, 2005; Schubert et al., 2013). In a recent study, Thomas and Pemstein (2015) found that the outcomes of social cooperation games could be biased in favor of one of the partners merely by providing visual cues from web cameras that showed one player to be above the other.

Further, these findings have important implications for work interactions, social interactions, and social-emotional development of those with physical disabilities, especially those confined to wheelchairs. Embodiment theory would suggest that those who do not have use of their body may not experience these emotions in the same way as non-disabled people and may not use the same visual cues to communicate social status emotions effectively. This may thereby contribute to well-known social challenges faced by those with disabilities. Such effects would likely have an impact not only on the social dynamic between individuals, influencing both the person producing the emotional display, but also on people observing that person’s display. For example, non-disabled individuals may over or under estimate the emotional experience of those with physical disabilities that may lead to more challenging interactions and affect hierarchical relations within work-place settings. There might be an increased likelihood that social status could be miscommunicated if one individual had a physical disability and the other did not. Also, if those constrained to wheelchairs violate expectations by not showing these nonverbal behaviors, people may make individual rather than situational attributions and perceive the person as less powerful or dominant.

Correspondingly, our findings support anecdotal reports of physically disabled individuals in wheelchairs feeling marginalized. Individuals with physical disabilities often report that non-disabled persons ignore them or do not afford them appropriate respect or status in social or work environments. Many people with physical disabilities who are confined to wheelchairs believe that they experience and communicate emotions well, but they also report being ignored and disrespected in social interactions as well as begin undervalued and subtly discriminated at work (Gay, 2004; Daley et al., 2018).

Given that this study was conducted with non-disabled individuals, a natural next step would be to follow up this study with physically disabled participants. It would be important to assess face vs. body use preferences for the different emotions among those who have experienced a movement restriction since birth as well as those who have acquired the restriction later in life. To the degree that the source of the preference is based on one’s own prior experience instead of a specific evolved response or caused by the immediate situation, then this preference should be less evident in those who have been paraplegic since birth, for example.

As with the preference measure, an important follow-up question would be to assess emotion production and recognition in physically disabled populations to determine whether the outcomes from this study are applicable to those chronically confined to a sitting position in a wheelchair. That is, for the participants in this study, was it the difference between their normal stature and ability to communicate that affected their display, or is there a more general effect such that those who are always restricted will show the same differences in display? Reports from actual wheelchair users indicate that they feel less able to express dominant emotions, particularly anger, while in a wheelchair (Cahill and Eggleston, 1994, p. 304). One woman, relegated to a wheelchair, was so angry she indicated that she wanted to jump out of her chair and shake the person she was directing her anger toward, but all she did was remain seated and “grit her teeth.” New wheelchairs that raise the individual to the eye level of non-disabled individuals (e.g., https://www.quantumrehab.com/ilevel-power-chairs/) may help reduce at least the relative height differences contributing to implicit status differences.

These findings suggest additional questions to be addressed with future research. Do people who have physical differences that constrain bodily movement alter their displays, especially for these target emotions (i.e., when the restriction is more chronic, have people learned to adjust displays of body-related emotions)? Do people who have physical differences that constrain bodily movement correspondingly adjust their confidence in expression of these emotions? Finally, do perceivers who have physical differences that constrain bodily movement more accurately perceive emotions (especially status related emotions) of others with similar physical differences? If so, this may be because the embodiment of these displays is consistent across expresser and the perceiver, or that given social-grouping differences, those with these physical differences have more experience interacting with and perceiving these displays. The answer to these questions may provide information on whether people’s display is more influenced by the broader social context, in which personal history would matter less or in sensitivity to changes in their own body.

Finally, we also want to consider perceiver effects, especially between non-disabled and disabled individuals. Do perceivers’ stereotypes influence what they see regarding emotional expression in people in a wheelchair? In other words, would perceptions be the same if the person were constrained in a wheelchair vs. a kitchen chair or a paraplegic person vs. hostage? It is possible that non-disabled perceivers stereotype people in wheelchairs as less dominant, and this affects the social dynamic between individuals. Studies examining the perception of wheelchair users by non-disabled individuals showed that non-disabled individuals associate more negative emotions (e.g., depression and guilt) with the disabled person (Vilchinsky et al., 2010). As with the higher status displays, those perceiving physically disabled people in wheelchairs may fail to account for the situational influences on emotional expression and may attribute the displays to the person’s internal state not situational constraint.

In conclusion, recent work investigating embodiment theory emphasizes that the current state of the body can have an important influence on psychological and emotional processes. Consistent with this, we found that constraints on body movement and position influence how emotions are produced and how they are perceived. This study’s findings provide only partial support for a pure embodiment perspective. Current body inputs do not appear to influence the expresser’s perception of their own emotional communication: people’s confidence in their displays did not change with body constraints. However, the results emphasize the important contributions of the body in nonverbal emotional communication and how these contributions may affect the social-emotional context of those with physical disabilities.

## Data Availability Statement

The datasets generated for this study are available on request to the corresponding author.

## Ethics Statement

The studies involving human participants were reviewed and approved by Claremont McKenna College IRB. The patients/participants provided their written informed consent to participate in this study.

## Author Contributions

CR was the project lead. KM, SA, and AS contributed parts of their independent research and thesis projects to the manuscript. EM and DM contributed expertise in the intellectual formation of the project and in writing the manuscript. All authors contributed to the article.

### Conflict of Interest

The authors declare that the research was conducted in the absence of any commercial or financial relationships that could be construed as a potential conflict of interest.

## Footnotes

^1^Consistent with the analyses conducted on the full data set, the group × face/body use × emotion ANOVA conducted with Huynh-Feldt corrected, equal sized groups found no main effects of group *F*(1,40) = 0.002, *p* = 0.96 and emotion *F*(4.95, 198.13) = 1.78, *p* = 0.12; no group × emotion interaction *F*(4.95, 198.13) = 1.03, *p* = 0.40; a main effect of face/body use *F*(1.26, 50.50) = 40.86, *p* < 0.0001; no group × face/body use interaction *F*(1.26, 50.50) = 2.69, *p* = 0.10; a significant emotion × face/body use interaction *F*(6.50, 259.93) = 12.36, *p* < 0.0001; and a significant 3-way interaction *F*(6.50, 259,93) = 2.11, *p* = 0.02.
